# *OsHAK1*, a High-Affinity Potassium Transporter, Positively Regulates Responses to Drought Stress in Rice

**DOI:** 10.3389/fpls.2017.01885

**Published:** 2017-11-01

**Authors:** Guang Chen, Chaolei Liu, Zhenyu Gao, Yu Zhang, Hongzhen Jiang, Li Zhu, Deyong Ren, Ling Yu, Guohua Xu, Qian Qian

**Affiliations:** ^1^State Key Lab for Rice Biology, China National Rice Research Institute, Hangzhou, China; ^2^State Key Laboratory of Crop Genetics and Germplasm Enhancement, MOA Key Laboratory of Plant Nutrition and Fertilization in Lower-Middle Reaches of the Yangtze River, Nanjing Agricultural University, Nanjing, China

**Keywords:** drought tolerance, *OsHAK1*, potassium homeostasis, rice (*Oryza sativa*), ROS

## Abstract

Drought is one of the environmental factors that severely restrict plant distribution and crop production. Recently, we reported that the high-affinity potassium transporter OsHAK1 plays important roles in K acquisition and translocation in rice over low and high K concentration ranges, however, knowledge on the regulatory roles of OsHAK1 in osmotic/drought stress is limited. Here, transcript levels of *OsHAK1* were found transiently elevated by water deficit in roots and shoots, consistent with the enhanced GUS activity in transgenic plants under stress. Under drought conditions, *OsHAK1* knockout mutants (KO) presented lower tolerance to the stress and displayed stunted growth at both the vegetative and reproductive stages. Phenotypic analysis of *OsHAK1* overexpression seedlings (Ox) demonstrated that they present better tolerance to drought stress than wild-type (WT). Compared to WT seedlings, *OsHAK1* overexpressors had lower level of lipid peroxidation, higher activities of antioxidant enzymes (POX and CAT) and higher proline accumulation. Furthermore, qPCR analysis revealed that *OsHAK1* act as a positive regulator of the expression of stress-responsive genes as well as of two well-known rice channel genes (*OsTPKb* and *OsAKT1*) involved in K homeostasis and stress responses in transgenic plants under dehydration. Most important, *OsHAK1*-Ox plants displayed enhanced drought tolerance at the reproductive stage, resulting in 35% more grain yield than WT under drought conditions, and without exhibiting significant differences under normal growth conditions. Consequently, *OsHAK1* can be considered to be used in molecular breeding for improvement of drought tolerance in rice.

## Introduction

Drought is one of the most widespread environmental condition that pose drastic decline of plants’ growth and crops’ productivity (Zhu, 2002). Throughout evolution, plants have acquired a series of strategies to avoid water deficit by diminishing water loss or increasing water uptake. Even so, other strategies are necessary to prevent cellular damage when water is exhausted and tissue dehydration is anticipated (Verslues et al., 2006).

Accumulation of reactive oxygen species (ROS) is distinctive under stress conditions including drought (Verslues et al., 2006). In plant cells, ROS such as hydrogen peroxide (H_2_O_2_), hydroxyl radicals and superoxide, are generated via aerobic metabolism, and as harmful oxygen derivatives, they can break lipids, nucleic acids, proteins, and carbohydrates, resulting in cellular damage and eventually cell death (Mittler et al., 2004). To reduce oxidative stress, organisms have evolved effective antioxidant defensive mechanisms which involve the induction of stress-related genes (Gasch et al., 2000; Desikan et al., 2002).

Maintenance of cellular homeostasis in plant cells is generally achieved through a ROS-scavenging system which is mainly assisted by enzymatic systems, such as catalase (CAT) and peroxidases (POXs) (Mittler et al., 2004). CAT breaks down H_2_O_2_, therefore, increased CAT activity results in lower cellular H_2_O_2_ levels (Scandalios, 2002). POXs utilize H_2_O_2_ to catalyze the oxidation of several substrates such as phenolic compounds (Asada, 1999), hence increased activity of these enzymes would decrease ROS levels. Recent studies have evidenced that transgenic rice plants with enhanced ROS-scavenging capacity present improved drought tolerance (Jiang et al., 2016).

Potassium (K) is the primary cation in plants, and affects all aspects of crop production including yield, resistance to pathogens and tolerance to abiotic stresses such as salinity, lodging, and drought (Ahmad et al., 2016b). K nutrition is closely related to water homeostasis and water use efficiency (Kuchenbuch et al., 1986; Tanguilig et al., 1987). An important response in drought-stressed plants is the uptake of solutes such as K (Andersen et al., 1992; Wang et al., 2004; Mahouachi et al., 2006). Limiting K loss supports osmotic adjustment, sustain cell expansion, ensures appropriate stomatal regulation and helps to sustain photosynthetic activity through photoassimilate translocation (Römheld and Kirkby, 2010; Zörb et al., 2014), therefore, modulation of K transport is crucial under stress conditions.

The putative function of the *KT/HAK/KUP* transporters has been predicted to play a key role in maintaining K homeostasis (Bañuelos et al., 2002; Gierth et al., 2005; Nieves-Cordones et al., 2007; Fulgenzi et al., 2008; Yang et al., 2014; Chen et al., 2015b; Li et al., 2017). In *Arabidopsis, KUP6* subfamily transporters may act as the key factors in osmotic adjustment by balancing K homeostasis in cell growth and drought stress (Osakabe et al., 2013). Perception of osmotic stresses can trigger the transient K effluxes at the plasma membrane by impairing HAK5 activity (Brauer et al., 2016). However, *KT/KUP/HAK* transporters have not been characterized in terms of affecting tolerance to osmotic or drought stress in other plant species (Li et al., 2017).

In our previous study, the expression pattern and physiological function of OsHAK1 in terms of K acquisition and transport in rice under various K and NH_4_^+^ supply conditions were intensively investigated. Results showed that knockout of *OsHAK1* led to growth retardation and decreased K accumulation irrespective of the K supply (Chen et al., 2015b), which led us to hypothesized that *OsHAK1* overexpression plants could exhibit higher K acquisition efficiency, a stronger growth phenotype and increased grain yield, especially when grown in adverse environmental conditions. Therefore, this work focused on the role of *OsHAK1* in drought stress responses. Our data indicates that changes in *OsHAK1* expression notably affect drought sensitivity, suggesting that this gene could offer advantages to breeding approaches for improving drought tolerance in crops.

## Materials and Methods

### Plant Materials and Growth Conditions

The generation and basic molecular properties of *OsHAK1* transgenic lines (*OsHAK1p:GUS, OsHAK1p:OsHAK1*[*OsHAK1*-Ox], and *oshak1* homozygous mutants in cv. Dongjin and cv. Manan genetic backgrounds) were previous described in Chen et al. (2015b).

For hydroponic experiments, seed sterilization and basal nutrient solution composition for seedling growth were described previously (Li et al., 2006). Same size 1-week-old rice seedlings were selected and transferred to IRRI nutrient solution (Chen et al., 2015b). The hydroponic experiments were carried out in a growth room with a 16 h light (30°C)/8 h dark (22°C) photoperiod and 70% relative humidity. In all treatments, nutrient solutions were replaced every 2 days. For drought stress experiments, rice seedlings were grown with the normal IRRI solution for 2 weeks and then transferred to nutrient solution supplemented with 15% (w/v) PEG6000 (mimics drought stress) for 1 week. At harvest, roots of rice plants were washed with 0.1 mM CaSO_4_ for 5 min. Roots and shoots were separated before recording their biomass and K concentrations were determined as described previously (Ding et al., 2006; Chen et al., 2015b).

Experiments with soil-grown plants were performed in a greenhouse. For assessing drought tolerance at seedling and reproductive stage, 5-week-old rice seedlings were grown in pots filled with 10 kg of air-dried loam soil until tillering or booting stage. Rice plants at tillering stage were divided into two groups for 3 weeks, while those at booting stage were divided into two groups until harvest: group 1 with full watering treatment as control and group 2 watered to approximately 40% of field capacity (Ahmad et al., 2016b). Each treatment included five biological replications.

### Gene Expression Analysis

Entire root and shoot tissues from WT and transgenic lines after control or PEG treatment under hydroponic condition and the first two leaves from plants used in the soil drought experiment were used for isolation of total RNA. qRT-PCR was performed according to the protocol described previously (Chen et al., 2015a). The *Ubq* gene was used as internal control to normalize all data and expression levels were calculated by using the 2^-ΔΔC_T_^ relative quantification method (Li et al., 2014). Primers used for qRT-PCR are listed in Supplementary Table S1.

### GUS Staining and Quantitative Measurement of GUS Activity

Histochemical GUS assay in different tissues from plants subjected to PEG treatment was carried out as described previously (Ai et al., 2009). Quantification of GUS activity was performed as described by Chen et al. (2015a). Intensity of 4-methylumbelliferone fluorescence was measured using a multi-mode microplate reader (SpectraMax M5, Molecular Devices, United States). Protein concentrations were determined using the Coomassie blue G-250 colorimetric assay.

### Measurement of Root Number and Length

The adventitious roots were scanned to record differences in the elongation of roots among the plant genotypes and treatments. The analysis was carried out as described previously (Song et al., 2011) using the WinRhizoV4.0b (Regent Instrument, Canada) root analysis system. A ruler was used to measure the length of adventitious roots. Five individual plants of each line were measured.

### Determination of Relative Water Content

Relative water content (RWC) was determined according to the method described by Zhao et al. (2014). Leaves were detached and weighted to obtain the fresh weight (FW) at the end of the stress period under soil treatment. Leaves were then soaked in de-ionized water for 4 h to obtain the saturated weight (SW). Subsequently, leaves were dried at 80°C for 48 h to determine dry weight (DW). RWC were calculated according to the formula: RWC = (FW - DW)/(SW - DW) × 100%.

### Electrolyte Leakage

Analysis of electrolyte leakage was carried out following the protocol of Guo et al. (2016). Fresh leaf samples from the soil treatment were harvested and washed with deionized water. Each sample was immediately placed into a beaker containing 30 mL of deionized water. The beaker was incubated at 25°C, shaken at 120 rpm for 3 h, and then electrical conductivity (EC1) of the solution was measured with a conductivity meter (Hanna, Italy). Next, samples with the immersion solution were boiled for 20 min and the conductivity measured after cooling it to room temperature (EC2). Relative electrolyte leakage (REL) was defined as REL = EC1/EC2 × 100%.

### Measurement of Photosynthetic Characteristics

Photosynthetic CO_2_ fixation rates were measured in rice seedlings between 9.00 and 11.00 am using a Li-COR6400 portable photosynthesis system equipped with a LED leaf cuvette (Li-COR, Lincoln, NE, United States), essentially as described in Li et al. (2016). At least five individual WT and transgenic lines in each stress treatment were selected for the measurements.

### Chlorophyll Concentration

Determination of chlorophyll concentration was performed following previously described procedures (Li et al., 2016). Leaves of WT and transgenic lines were harvested, weighed and extracted with aqueous ethanol (95% v/v). The absorbance (A) of the extract was recorded at wavelengths of 663 and 645 nm using a spectrophotometer (Shimadzu UV2400, Japan). Total chlorophyll concentration was calculated as 8.02A_663_+20.21A_645_, and was expressed as mg chlorophyll g^-1^ FW.

### Proline Content

Proline content in rice leaves was determined according to the method described by Bates et al. (1973). About 0.5 g of leaf tissue was homogenized in 5 mL of 3% sulfosalicylic acid. After centrifugation at 12,000 × *g* for 10 min, the supernatant (1 mL) was mixed with 1 mL of ninhydrin and 1 mL of glacial acetic acid and then incubated at 100°C for 1 h. The reaction was then cooled down in an ice bath. Two milliliters of toluene was added to extract the resulting colored product and the absorbance was measured at 520 nm with a microplate reader (SpectraMax M5).

### Determination of Malondialdehyde (MDA) Content

Malondialdehyde (MDA) content was determined based on the method described by Heath and Packer (1968) and described in detail in Cai et al. (2015), with minor modifications. Briefly, approximately 0.5 g of rice leaves were homogenized in 10 mL of 10% trichloroacetic acid (w/v) and centrifuged at 5,000 × *g* for 10 min. The supernatant (2 mL) was reacted with 2 mL of a chilled mix of 0.6% (w/v) thiobarbituric acid in 10% (w/v) trichloroacetic acid in a test tube at 100°C for 15 min. The reaction was quickly cooled on ice and then centrifuged at 5,000 × *g* for 10 min. Absorbance of the supernatant was measured at 450, 532, and 600 nm and the MDA content was calculated using the equation: 6.45 × (OD_532_ - OD_600_) - 0.559 × OD_450_.

### Quantification of H_2_O_2_

Extraction and determination of hydrogen peroxide (H_2_O_2_) through reaction with 0.1% TiCl_4_ in 20% H_2_SO_4_ were performed according to the method of Mostofa and Fujita (2013).

### POX and CAT Activity

Total protein from rice leaves was extracted with 0.05 M potassium phosphate buffer (pH 7.0). After centrifugation at 12,000 × *g* at 4°C for 15 min, the resulting supernatant was used to determine POX and CAT activities. POX activity was determined according to the method described by Ning et al. (2010). Briefly, each POX reaction mixture contained 0.1 mL of the enzyme extract, 2.9 mL of 0.05 M potassium phosphate buffer (pH 5.5), and 1 mL of 0.5% (v/v) H_2_O_2_ plus 1 mL of 0.05 M guaiacol as substrates. Absorbance readings at 470 nm were performed every 10 s to monitor the oxidation of guaiacol. CAT activity was determined following the method reported by Mostofa and Fujita (2013).

### Statistical Analysis

Data were analyzed by ANOVA using the statistical SPSS 10 program (SPSS Inc., Chicago, IL, United States). Statistically differences (*P* ≤ 0.05) between WT and *OsHAK1* transgenic lines are indicated on the histograms by asterisks.

## Results

### Response of *OsHAK1* Gene Expression to Osmotic Stress

**FIGURE 1:**
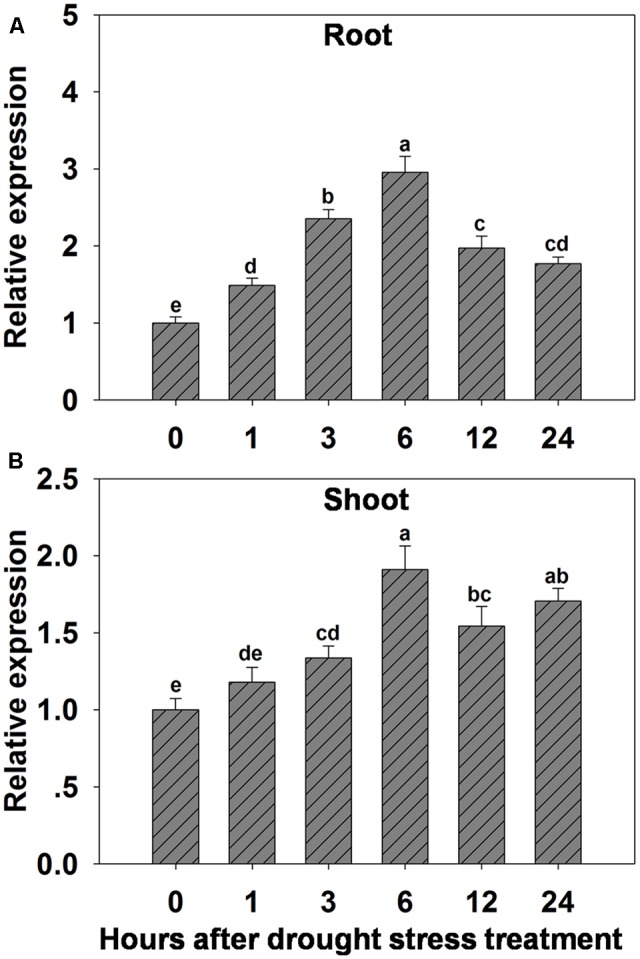
Effect of osmotic stress on the expression of *OsHAK1* in wild type rice plants. **(A,B)** Expression of *OsHAK1* under osmotic stress treatment in WT roots **(A)** and shoots **(B)**. Rice seedlings were supplied with normal IRRI solution for 14 days, then transferred to nutrient solution containing 15% PEG for different time (0, 1, 3, 6, 12, and 24 h). Total RNA were extracted from roots and shoots of rice cv. Nipponbare. *Ubq* was used as an internal control. The expression level for 0 h treatment was set to 1. Error bars indicate SE (*n* = 3). Bars with different letters are significantly different at *P* < 0.05.

The possibility that expression of *OsHAK1* could be affected by water deficit was analyzed by qRT-PCR. Results showed that expression of *OsHAK1* was significantly induced in both roots and shoots of plants treated with 15% PEG (**Figure 1**). Overall, *OsHAK1* was more induced in roots than in shoots after dehydration treatment. The expression profile of *OsHAK1* in both roots and shoots followed a sigmoid curve. In roots transcript levels increased after 1 h of PEG application, reaching its peak after 6 h, to then gradually decline with prolonged treatment (12–24 h), showing two-fold to three-fold increase (**Figure 1A**). In shoots, expression of *OsHAK1* was highly expressed at 6 h after treatment to later gradually decline after up to 12 h of stress, showing a barely two-fold increase (**Figure 1B**).

**FIGURE 2:**
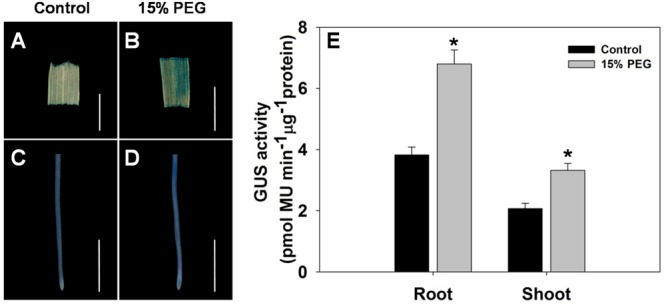
GUS activity in roots and shoots of *HAK1p:GUS* transgenic plants under control and stress conditions. **(A–D)** GUS staining in the leaf blade **(A,B)** and root **(C,D)** of *OsHAK1p:GUS* transgenic rice seedlings under normal and 15% PEG treatment. Transgenic rice seedlings were grown in normal IRRI solution for 2 weeks and then transferred to nutrient solution containing 15% PEG for 3 days. **(E)** Quantification of GUS activity. **(A,B)** Bars = 5 mm. **(C,D)** Bars = 2 mm. Error bars indicate SE (*n* = 3). Significant differences with the controls are indicated with asterisks (*P* < 0.05, one-way ANOVA).

To further evaluate the stress-regulated expression of *OsHAK1*, transgenic rice plants expressing the GUS reporter gene under the control of the *OsHAK1* promoter were generated. *OsHAK1* expression was examined in the roots and leaf blades (**Figures 2A–D**). GUS activity was significantly increased in *OsHAK1p: GUS* transgenic plants treated with 15% PEG (**Figure 2E**). Quantified GUS activity was also higher in roots than in shoots (**Figure 2E**), which was consistent with the qRT-PCR analysis (**Figure 1**). These results indicate that *OsHAK1* is induced by osmotic stress, and may be playing significant roles in the response of rice to drought.

### Effect of *OsHAK1* Expression on Rice Growth at Seedling Stage under Osmotic Stress

To understand any role of *OsHAK1* in drought stress responses, plants overexpressing this gene in cv. Nipponbare (Ox lines) and two T-DNA insertion lines of *oshak1* in the Dongjin and Manan backgrounds (KO lines) were obtained. Generation and basic characterization of *OsHAK1* transgenic lines have been previously described by Chen et al. (2015b).

**FIGURE 3:**
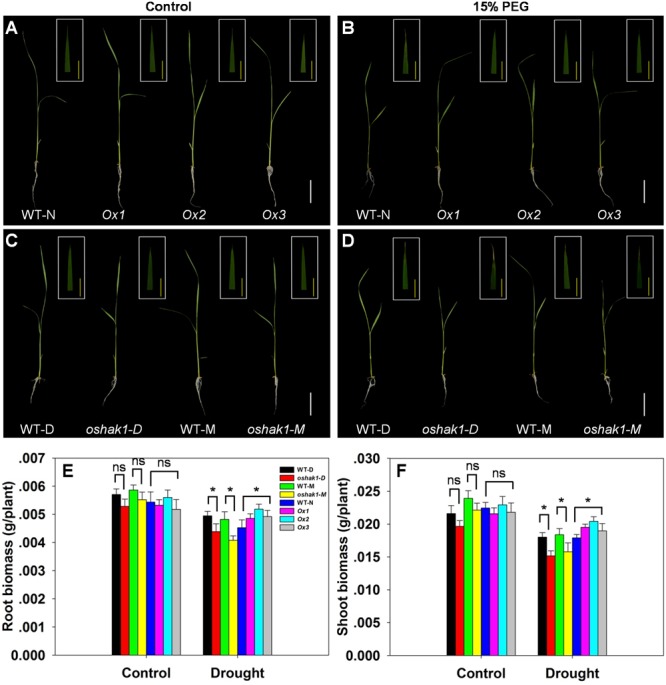
Effect of *OsHAK1* expression on rice growth at seedling stage under control and stress conditions. Rice seedlings were grown in normal IRRI solution for 2 weeks and then transferred to nutrient solution containing 15% PEG for 7 days. **(A,B)** Growth performance of Ox lines and their respective WT line and photographs of leaves of seedlings under control **(A)** and 15% PEG treatment **(B)**. **(C,D)** Growth performance of KO lines and their respective WT and photographs of leaves from seedlings under control **(C)** and 15% PEG treatment **(D)**. **(A–D)** White bars = 5 cm, Yellow bars = 1 cm. **(E,F)** Root **(E)** and shoot **(F)** biomass (dry weight) of plants grown in the conditions mentioned in **(A–D)**. The values are means ± SE of five replicates. Significant differences between each genotype and its respective wild type are indicated with asterisks (*P* < 0.05, one-way ANOVA); ns indicates non-significant differences at that level of significance. WT-NB: wild type of the Nipponbare cultivar. Ox1–Ox3: three independent lines of OsHAK1-overexpressing plants of the Nipponbare cultivar. WT-D: wild type of the Dongjin cultivar, *oshak1-D*: OsHAK1 knockout mutant line of the Dongjin cultivar. WT-M: wild type of the Manan cultivar, *oshak1-M*: OsHAK1 knockout mutant line of the Manan cultivar.

To further assess how *OsHAK1* expression affects rice growth, WT, KO, and Ox lines were grown hydroponically in control medium and solution with 15% PEG to produce an osmotic stress treatment mimicking drought stress. As shown in **Figures 3A,C**, no differences were detected between the growth of each genotype and its respective wild type when they were grown under control condition. Upon exposure to the dehydration medium, wilting and foliar chlorosis was observed in WT and KO lines, but not in the *OsHAK1* overexpressing plants (**Figures 3B,D**). In addition, the stress treatment led to suppression of shoot and root growth in all three genotypes. The magnitude of reduction of shoot and root growth in Ox plants was significantly less than that in KO plants (**Figures 3A–D**). Plant height, dry root biomass, and shoot biomass of Ox seedlings were significantly higher than those of WT under stress condition. Meanwhile, the total dry weights of the *oshak1* mutants grown with 15% PEG decreased in average to about 87% of their respective WT (**Figures 3E,F**).

**FIGURE 4:**
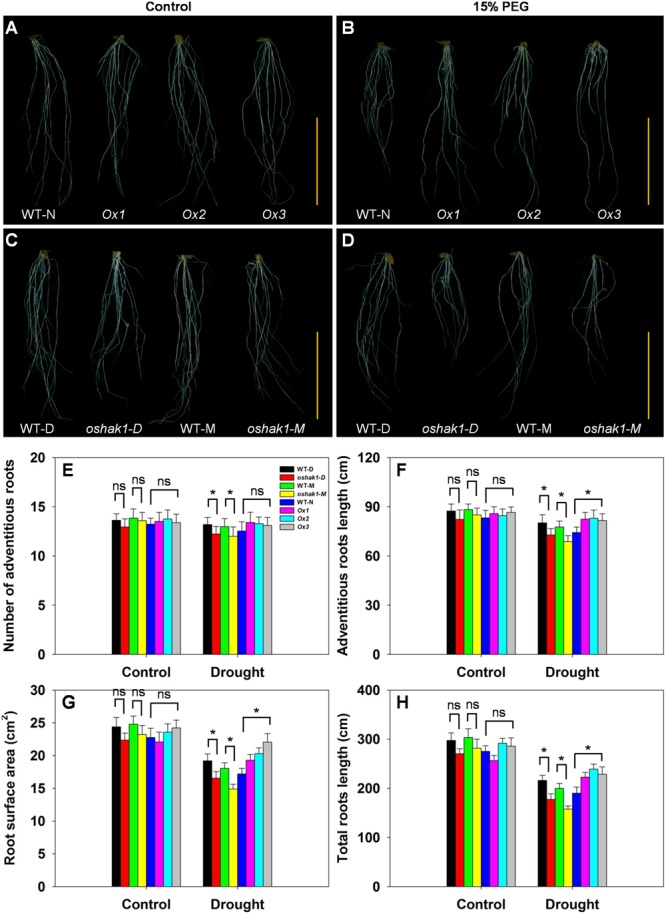
Effect of *OsHAK1* expression on root system architecture at seedling stage under control and stress conditions. Rice plants were grown under the conditions described in the legends of **Figure 3**. **(A,B)** Root phenotypes of Ox lines and their respective WT line under control **(A)** and 15% PEG treatment **(B)**. **(C,D)** Root phenotypes of KO lines and their respective WT lines under control **(C)** and 15% PEG treatment **(D)**. **(A–D)** Bars = 5 cm. **(E–H)** Adventitious root number **(E)**, adventitious root length **(F)**, root surface area **(G)**, and total root length **(H)** of plants grown in the conditions mentioned in **(A–D)**. The values are means ± SE of five replicates. Significant differences between each genotype and its respective wild type are indicated with asterisks (*P* < 0.05, one-way ANOVA); ns indicates non-significant differences at that level of significance.

The architecture of the root system is an important trait responsible for efficient nutrient acquisition under stress conditions. The observation that *OsHAK1* expression affected rice growth under stress conditions, prompted us to evaluate whether the roots of *OsHAK1* transgenic lines differed from WT in their response to the stress treatment. As shown in **Figure 4**, exposure of the seedlings to dehydration medium led to significant reductions in their adventitious root length, root surface area, and total root length. The reduction was greater in KO lines than in their respective WT (**Figure 4**), such that KO lines showed shortened length of the total root system and adventitious roots, and decreased adventitious root number and root surface area. However, these parameters maintained the opposite tendency in the Ox lines (**Figures 4E–H**). These results suggest that the larger root system of Ox lines under dehydration stress may facilitate nutrition acquisition, thus providing beneficial conditions for rice growth and water stress tolerance.

### Effect of *OsHAK1* Expression on K and Na Homeostasis at Seedling Stage under Osmotic Stress

**FIGURE 5:**
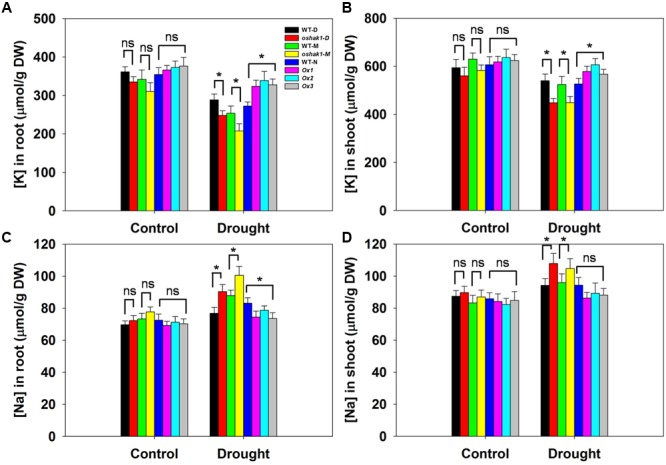
Effect of *OsHAK1* expression on K and Na accumulation at seedling stage under control and stress conditions. Rice seedlings were cultured as described in **Figure 3**. **(A,B)** K concentrations in roots **(A)** and shoots **(B)** of plants under control and 15% PEG treatment. **(C,D)** Na concentrations in roots **(C)** and shoots **(D)** of plants under control and 15% PEG treatment. Data represent mean ± SE of five replicates. Significant differences between each genotype and its respective wild type are indicated with asterisks (*P* < 0.05, one-way ANOVA); ns indicates non-significant differences at that level of significance. DW, dry weight.

Given that the improved stress tolerance of *OsHAK1*-Ox plants may be due to increased K accumulation in root and shoot tissues, moreover, Na competes with K for uptake across the plasma membrane, we compared the effects of the osmotic stress on K and Na concentrations in roots and shoots. There were no significant differences between each genotype and its respective WT under control conditions (**Figure 5**). In the presence of 15% PEG, KO lines had extremely low K concentration while Ox lines contained significantly more K in both root and shoot tissue (1.26-fold in roots and 1.17-fold in shoots) than their respective WT (**Figures 5A,B**), whereas Na content maintained the opposite tendency (**Figures 5C,D**). These results suggested that sustained high K levels in *OsHAK1* overexpression rice plants can contribute to the enhanced tolerance to water deficit.

**FIGURE 6:**
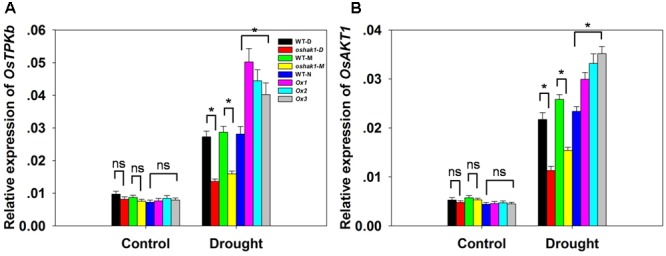
Expression level of genes encoding K channels in *OsHAK1* transgenic lines and the respective wild types. Plants were grown as described in the legend of **Figure 3**. RNA was extracted from roots and qRT-PCR was used to detect the transcript level of *OsTPKb* **(A)** and *OsAKT1* **(B)**. PCR signals were normalized with *Ubq* transcripts. Data are means ± SE of three biological replicates. Significant differences between each genotype and its respective wild type are indicated with asterisks (*P* < 0.05, one-way ANOVA); ns indicates non-significant differences at that level of significance.

To better understand the mechanisms underlying K accumulation in *OsHAK1* transgenic seedlings, we analyzed the expression level of genes encoding K transport proteins. The data showed that drought enhanced the expression of *OsTPKb* (K selective vacuolar channel) and *OsAKT1* (K inward rectifying channel) in all genotypes (**Figures 6A,B**). In addition, these genes were the most strongly induced under osmotic stress in overexpression seedlings compared to KO lines and WT (**Figure 6**). In contrast, the expression level in KO lines was evidently lower than in WT (**Figure 6**).

### *OsHAK1* Transgenic Plants Present Improved Physiological Parameters under Drought Stress

To evaluate plants’ response to drought in a more natural growing condition, rice seedlings grown hydroponically were transferred to pots to further investigate the phenotype of all three genotypes applying full watering (100% field capacity) or drought stress (40% field capacity) for a period of 3 weeks. The *oshak1* mutants took up substantially less K into both their roots and shoots under both control and drought stress conditions, and the over-expressor plants accumulated significantly more K in both their root and shoot tissue than did WT plants (Supplementary Figure S1).

**FIGURE 7:**
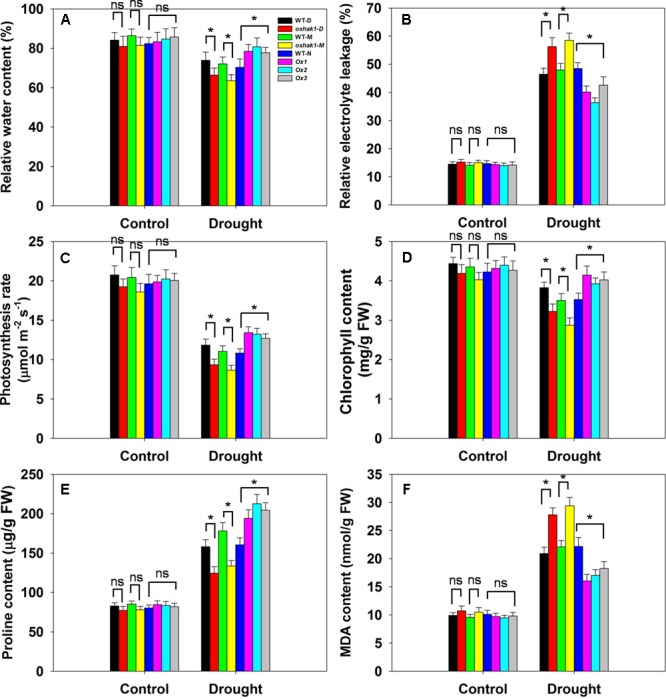
Physiological parameters of *OsHAK1* transgenic lines and their respective wild types. Five-week-old rice plants were transferred to soil pots and exposed limited water supply (40% field capacity) or full watering (100% field capacity) as control for additional 3 weeks. Relative water content **(A)**, relative electrolyte leakage **(B)**, photosynthesis rate **(C)**, chlorophyll content **(D)**, proline content **(E)**, and MDA content **(F)** were assayed. Values are shown with mean ± SE (*n* = 5). Significant differences between each genotype and its respective wild type are indicated with asterisks (*P* < 0.05, one-way ANOVA); ns indicates non-significant differences at that level of significance. FW, fresh weight.

Several physiological parameters including RWC, REL, photosynthesis rate (Pn), chlorophyll content, proline content, and MDA content were examined in the transgenic lines and the respective WT plants to assess whether any differences can be associated with a higher tolerance to drought. Under normal growth condition, no significant differences in leaf RWC was observed between transgenic lines and WT plants (**Figure 7A**). When subjected to drought stress, RWC of both WT and transgenic lines was reduced; however, RWC was noticeably higher in the Ox lines and lower in the KO lines compared to the WT plants (**Figure 7A**). Under control conditions, electrolyte leakage, an indicator of membrane damage, was similar in transgenic and WT plants, whereas leaves of Ox lines presented significantly lower (20–25% decrease) electrolyte leakage levels compared to WT after 3 weeks of stress treatment (**Figure 7B**). Moreover, over 60% of the ions leaked from tissue of KO plants, while ion leakage of their respective WTs was less than 50% (**Figure 7B**).

Photosynthesis rate and chlorophyll content are important biochemical trait markers of stress tolerance in plants. We compared the effects of drought stress on Pn and foliar chlorophyll content of the three genotypes. Both Pn and chlorophyll content were comparable in the three genotypes under control conditions (**Figures 7C,D**). After drought treatment, there were significant reductions in Pn in all genotypes, but the magnitude of the decline was greater in WT than in Ox plants, leading to a significantly higher Pn in Ox lines (**Figure 7C**). Similarly, a significant reduction in leaf chlorophyll content was detected in WT seedlings, while Ox plants maintained relatively constant chlorophyll content when challenged by drought (**Figure 7D**). On the other hand, the KO lines exhibited dramatically lower Pn and chlorophyll content than their respective WT cultivars under drought conditions (**Figures 7C,D**).

The accumulation of compatible osmolytes such as proline (Pro), is generally, considered to be an adaptive response against environmental stresses (Ahmed et al., 2013). Meanwhile, accumulation of MDA content reflects damage to the structural integrity of cell membranes caused by oxidative stress such as that derived from drought stress (Zhao et al., 2014; Cai et al., 2015). To associate these functional attributes with the apparent drought tolerance exhibited by *OsHAK1* overexpressors, Pro and MDA contents were analyzed. Accordingly, Pro and MDA contents in transgenic lines were similar to those in WT plants when grown under control condition (**Figure 7E**). Drought treatment resulted in an overall increase in Pro content in both transgenic and WT plants compared to those under control condition (**Figure 7E**). Even so, the *OsHAK1* Ox lines exhibited 27% more Pro content after 3 weeks of drought stress compared to WT plants, whereas in KO lines Pro accumulation was about 25% less than that in WT plants after drought treatment (**Figure 7E**). In contrast, *OsHAK1* overexpression caused significant reduction in the accumulation of MDA. Under drought stress, Ox plants had 23% less MDA compared to WT plants (**Figure 7F**), meanwhile KO plants presented 33% additional MDA content than WT (**Figure 7F**).

### *OsHAK1* Transgenic Plants Have Altered ROS-Scavenging Capacity

**FIGURE 8:**
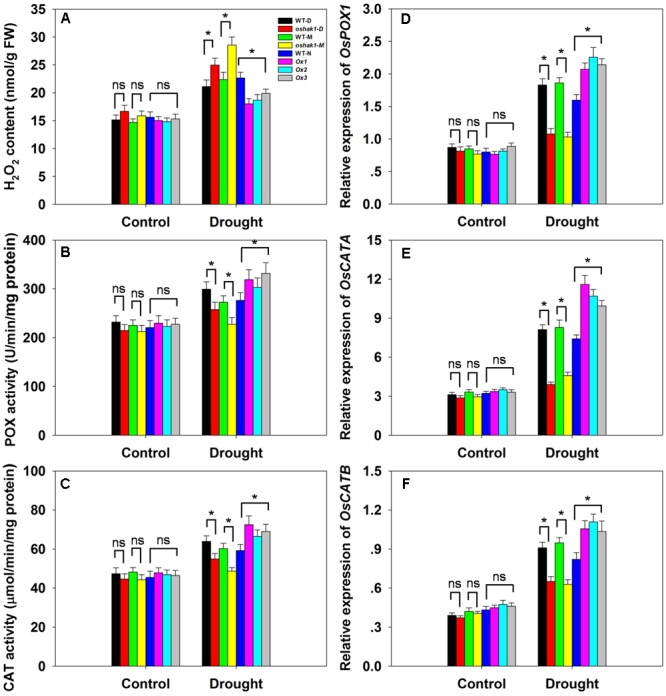
Effect of *OsHAK1* expression on ROS-scavenging capacity. Growth conditions and treatments were the same as described in **Figure 7**. **(A–C)** Physiological indexes reflecting ROS-scavenging capacity under normal and drought stress: H_2_O_2_ content **(A)**, enzymatic activities of POX **(B)**, and CAT **(C)**. The values are means ± SE of five replicates. Expression level of *OsPOX1* **(D)**, *OsCATA* **(E)**, and *OsCATB* **(F)** were tested by qRT-PCR. Total RNA was isolated from leaf blades. *Ubq* was used as a control. Data are means ± SE of three biological replicates. Significant differences between each genotype and its respective wild type are indicated with asterisks (*P* < 0.05, one-way ANOVA); ns, non-significant differences at that level of significance. FW, fresh weight.

Drought usually causes injury at the cellular level via oxidative stress including the generation of ROS, such as H_2_O_2_ and superoxide (Mittler, 2002; Xiong and Zhu, 2002; Jiang et al., 2016). The lower level of lipid peroxidation in Ox transgenic plants, indicated by a reduced MDA accumulation, may be associated with a reduced ROS accumulation upon drought stress. Therefore, we determined whether *OsHAK1* is involved in ROS detoxification. Indeed, the Ox plants showed much less drought-induced H_2_O_2_ accumulation compared to WT (**Figure 8A**). In contrast, H_2_O_2_ accumulation in KO lines was ∼23% higher compared to their respective WT, while both WT and transgenic plants showed similar H_2_O_2_ levels under control conditions (**Figure 8A**). The reduced stress-induced H_2_O_2_ accumulation in the Ox lines could be the result of changes in ROS-scavenging activities such as those of POX and CAT, enzymes involved in H_2_O_2_ elimination (Mittler, 2002; Apel and Hirt, 2004; Cai et al., 2015). Under normal growth conditions, levels of POX and CAT activities were not significantly different among the genotypes tested, however, after 3 weeks of drought treatment, both activities were markedly higher in Ox plants and lower in the KO lines compared to WT plants (**Figures 8B,C**). We further assayed the expression of genes encoding for *OsPOX1, OsCATA*, and *OsCATB*. Consistent with the increase of the enzymes activities, all three genotypes presented up-regulation of the transcript levels of these genes in response to drought, with a greater increase in Ox plants and a smaller increase in KO lines than in WT (**Figures 8D–F**). These results suggest that over-expression of *OsHAK1* enhances the ROS-scavenging capacity, which decreases ROS damage under drought stress conditions.

### *OsHAK1* Transgenic Plants Present Altered Expression of Stress-Responsive Genes under Drought Conditions

**FIGURE 9:**
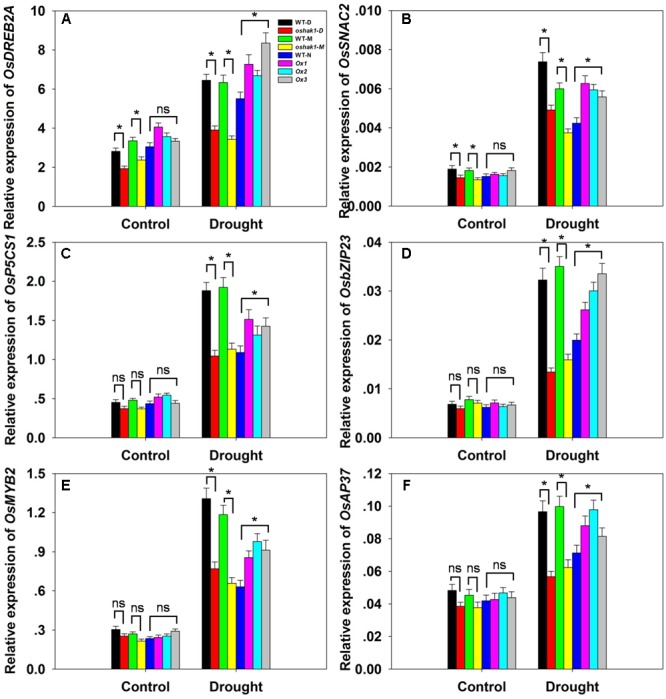
. Expression of stress-responsive genes in *OsHAK1* transgenic lines. Growth conditions and treatments were the same as described in **Figure 7**. RNA was extracted from leaf blades and qRT-PCR was used to detect the transcript level of *OsDREB2A* **(A)**, *OsSNAC2* **(B)**, *OsP5CS1* **(C)**, *OsbZIP23* **(D)**, *OsMYB2* **(E)**, and *OsAP37* **(F)**. PCR signals were normalized with *Ubq* transcripts. Data are means ± SE of three biological replicates. Significant differences between each genotype and its respective wild type are indicated with asterisks (*P* < 0.05, one-way ANOVA); ns, non-significant differences at that level of significance.

To further understand the mechanism underlying the increased drought tolerance in *OsHAK1*-overexpressing plants, we performed gene expression profiling of selected genes known to respond to multiple abiotic and biotic stresses. These were *OsDREB2A* (Dubouzet et al., 2003), *SNAC2* (Nakashima et al., 2007; Hu et al., 2008), *OsP5CS1* (Sripinyowanich et al., 2013), *OsbZIP23* (Zong et al., 2016), *OsMYB2* (Yang et al., 2012), and *OsAP37* (Oh et al., 2009). Under normal growth conditions, most genes displayed relatively weak expression and insignificant variations of transcription levels in both WT and transgenic plants except for *OsDREB2A* and *SNAC2*, which were significantly down-regulated in KO lines (**Figures 9A–F**). After 3 weeks of stress treatment, the relative expression of the genes was notably increased (**Figures 9A–F**). Stress-related transcription factors genes such as *OsDREB2A, SNAC2, OsbZIP23, OsMYB2*, and *OsAP37* were up-regulated in Ox plants (0.25- to 1-fold over WT plants) after drought treatment (**Figures 9A,B,D–F**). Expression of the delta-pyrroline-5-carboxylate synthetase gene, *OsP5CS1*, was strongly induced in Ox lines under drought stress compared to control plants (**Figure 9C**). The higher transcript level of *OsP5CS1* was consistent with the higher Pro content in Ox plants (**Figure 7E**). By contrast, expression of all of these genes showed opposite trends in *oshak1* mutants, with noticeably lower values when compared to WT plants subjected to drought (**Figures 9A–F**). These data indicate that over-expression of *OsHAK1* in rice enhances the expression of a set of stress-response genes that ultimately can support an increased tolerance to drought.

### Overexpression of *OsHAK1* Significantly Improves Drought Resistance at Reproductive Stage

**FIGURE 10:**
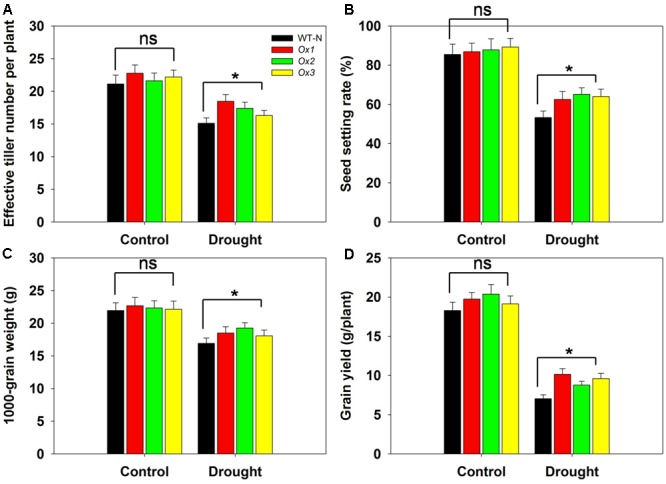
Overexpression of *OsHAK1* improves drought resistance at reproductive stage. Rice plants were grown in pots and were fully irrigated by watering every day until the drought treatment. Drought stress was applied at the booting stage. Limited water supply (40% field capacity) was maintained throughout the experiment until completion of the life cycle and full watering was applied to controls, then agronomic traits were assayed. **(A)** Effective tiller number per plant. **(B)** Seed-setting rate. **(C)** 1000-seed grain weight. **(D)** Grain yield per plant. Data represent mean ± SE of five replicates. Significant differences between WT and *OsHAK1*-Ox lines are indicated with asterisks (*P* < 0.05, one-way ANOVA); ns, non-significant differences at that level of significance.

Reports have demonstrated that rice yields are affected by drought stress at the booting stage (Guan et al., 2010; Zhang et al., 2013). To evaluate whether *OsHAK1* can function in improving rice yield under stress conditions at this developmental stage, we compared several agronomic traits between *OsHAK1*-Ox and WT plants. T_5_ transgenic Ox lines and WT plants were subjected to 40% field capacity treatment at the booting stage until harvest. Results showed that all *OsHAK1* Ox plants exhibited more effective tiller number (more than 10–15%) (**Figure 10A**), higher spikelet fertility (15–20% higher) (**Figure 10B**), increased 1000-grain weight (more than 7–10%) (**Figure 10C**), and higher grain yield per plant (25–35% higher) (**Figure 10D**) compared to WT under drought conditions. Meanwhile, under well irrigation, all Ox lines and WT plants displayed similar performance for these agronomic traits (**Figures 10A–D**). These results indicate that over-expression of *OsHAK1* did not affect growth or yield productivity of rice grown under normal conditions, but improved the degree of drought tolerance under water-limiting conditions.

## Discussion

Predicted functions of plants’ KT/HAK/KUP transporters include maintaining K and Na homeostasis under low K and high salt conditions. In rice, OsHAK5 plays a role in root K acquisition under low external K concentrations and in K upward transport from roots to shoots (Yang et al., 2014). OsHAK1 has been defined to be essential for maintaining K-mediated growth and to play important roles in K acquisition and transport within plant organs over a wide range of K concentrations (Chen et al., 2015b). Still, few reports have deciphered the mechanisms by which KT/HAK/KUP transporters could participate in drought stress responses. Data in this study revealed that OsHAK1 contributes to drought stress tolerance. Supporting this, are these observations: (1) *OsHAK1* expression is induced by osmotic/drought stress; (2) changes in *OsHAK1* expression visibly affect drought stress tolerance in rice at the seedling and tillering stages; (3) *OsHAK1* regulates root system architecture under stress conditions that positively affects resistance to water deficit; (4) *OsHAK1* expression improves ROS-scavenging in rice plants. (5) Several stress-related genes are regulated by the expression of *OsHAK1*. (6) Overexpression of *OsHAK1* significantly improves crop productivity under drought conditions.

### *OsHAK1* Regulates K Homeostasis, Root and Shoot Growth to Enhance Drought Stress Resistance

Potassium nutrition is closely related to plant water homeostasis and water use efficiency (Kuchenbuch et al., 1986; Tanguilig et al., 1987; Ahmad et al., 2016a). Enhanced K uptake is a key response of plants suffering from drought (Andersen et al., 1992; Wang et al., 2004; Ahmad et al., 2016b) as limiting K deficiency improves water retention, ensures appropriate stomatal regulation and helps to maintain photosynthetic activity via photoassimilate translocation (Römheld and Kirkby, 2010; Zörb et al., 2014). A causal link between *OsHAK1* overexpression and improved tolerance to water deficit derives from the observation of a higher K accumulation in both roots and shoots of Ox plants compared to control plants (**Figures 5A,B** and Supplementary Figure S1). Improved K retention lowers cellular water potential and prevents further water loss, for example from roots to soil. As previously reported, OsTPKb can alter K concentration in small vacuoles, which determines the overall cellular K homeostasis which, in turn, influences stress tolerance (Ahmad et al., 2016a). It has been reported that overexpression of *OsAKT1* in rice improves osmotic and drought stress tolerance by boosting tissue K levels, particularly in roots (Ahmad et al., 2016b). To better comprehend the mechanisms of drought tolerance conferred by the relatively higher K levels in Ox lines, the expression of two reported genes were investigated. Significant up-regulation of the transcript levels of *OsTPKb* and *OsAKT1* was observed in *OsHAK1*-Ox plants compared to WT plants under osmotic stress conditions (**Figures 6A,B**), indicating that *OsHAK1* affected the expression of genes encoding K transporters or channels, such as *OsTPKb* and *OsAKT1*, that can stimulate drought tolerance in rice.

We observed that overexpression of *OsHAK1* driven by its native promoter significantly increased the growth of roots and aerial parts, whereas *OsHAK1* knockout lines displayed impaired growth compared to WT cultivars under stress (**Figures 3, 4**). In plants, K participates in maintaining both root and shoot growth, including regulation of cell cycle (Sano et al., 2007) and in the completion of cell death programs (Peters and Chin, 2007). We found that K concentration was markedly higher in *OsHAK1*-Ox plants and lower in the KO mutants compared to WT after water-limiting treatments under both hydroponic (**Figures 5A,B**) and soil culture conditions (Supplementary Figures S1A,B).

Characteristics of the root system are important for efficient K acquisition in plants growing under K-deficient conditions. In an *Arabidopsis* mutant of the HAK-homolog TRH1 (AtKUP4/AtKT3), both Rb^+^ uptake and root hair growth were inhibited, implying that TRH1-mediated K uptake is required for elongation of root hair cells (Rigas et al., 2001). *athak5* mutants presented significant shorter roots than those of WT when grown under low K levels or in the absence of the nutrient, however, such difference in growth was slim under a high external K medium (Qi et al., 2008). The observation that *OsHAK1* KO seedlings had a lower K concentration in their roots and shoots than WT plants under stress conditions, prompted us to evaluate whether their root systems differed in response to the treatments. The *oshak1* mutants showed shorter roots, dwarf size, less adventitious root number, and lower root surface area compared to WT under stress conditions at the seedling stage (**Figure 4**). In contrast, Ox lines showed an opposite trend with significantly improved root growth when plants were exposed to 15% PEG (**Figure 4**). These results suggest that the more extensive root system of Ox seedlings may facilitate K acquisition, thus equipping them to better overcome the K deficiency associated with drought stress.

Improved growth and yield productivity were observed in Ox lines at the harvest stage in soil water deficit experiments (**Figure 10**). There are two possible explanations for the phenotype of Ox plants. First is the enhanced vegetative and reproductive growth by increased K acquisition in the Ox lines. Overexpression of *OsHAK1* increased root surface and total root length which would lead to an improved absorption surface for K, hence, an increase of K concentrations in all the tissues when grown under stress conditions (**Figures 4G,H, 5A,B** and Supplementary Figure S1). The second is the enhanced chlorophyll content and photosynthetic rates in *OsHAK1* overexpressors under stress conditions. Chlorophyll content and photosynthetic rates in Ox lines were noticeably higher than in WT plants under drought stress (**Figures 7C,D**). *OsHAK1* led to decreased chlorophyll loss which benefits in sustaining photosynthetic rates, therefore helping rice adaptation to drought. These findings highlight that maintenance of a higher K concentration and a better growth performance, particularly with a larger root system, are influential strategies for tolerance to dehydration stress in rice.

### *OsHAK1* Decreases ROS Damage and Enhances ROS-Scavenging Capacity under Drought

Reactive oxygen species accumulation associated with abiotic and biotic stress responses is often considered a manifestation of stress-induced damage, but it can also have a role as signal to trigger stress adaptation (Dat et al., 2000). The experiments performed to evaluate oxidative damage, ROS accumulation and detoxification activities in *OsHAK1* transgenic lines and WT plants support two conclusions: (1) *OsHAK1*-Ox plants display fewer indications of oxidative stress upon challenge by dehydration and (2) *OsHAK1*-Ox plants activate detoxification mechanisms more readily when challenged by drought stress.

The first indication of oxidation damage was the lower MDA level detected in Ox lines under drought conditions compared to WT (**Figure 7F**). A similar trend was reported by Zhao et al. (2014), where MDA accumulation was significantly reduced in *Zmhdz10* overexpression plants (less sensitive to drought stress). Another sign of oxidative stress is H_2_O_2_ buildup. Accumulation of H_2_O_2_ as result of dehydration damage has been documented in rice (Cai et al., 2015; Jiang et al., 2016). In accordance with the lower MDA accumulation in *OsHAK1* Ox lines (**Figure 7F**), lower levels of H_2_O_2_ was also detected in these plants compared to WT under drought stress (**Figure 8A**).

Proline accumulation, generally considered an osmoprotection response associated with membrane and protein stability (Xiang et al., 2007; Zhao et al., 2014), was also measured. Although still debatable, several studies in rice support the idea that Pro functions in the osmotic adjustment under drought stress (Cai et al., 2015; Hong et al., 2016; Jiang et al., 2016). In our research, *OsHAK1*-Ox plants under drought conditions accumulated significantly more Pro compared to WT (**Figure 7E**). *P5CS1*, responsible for catalyzing Pro biosynthesis, is critical for increasing abiotic stress tolerance (Zhu et al., 1998). Drought, salt, and abscisic acid induce the expression of *OsP5CS1*, resulting in increased Pro content, and overexpression of *OsP5CS1* improves osmotolerance (Igarashi et al., 1997; Zhu et al., 1998). The distinctly elevated transcript levels of *OsP5CS1* were consistent with the high Pro content detected in Ox plants after drought treatment (**Figures 7E, 9C**). This increased Pro content could account for higher osmolarity, thus leading to a lower water potential and making plant tissues more efficient at retaining water (Song et al., 2012). Consistently, the leaves of Ox lines presented superior RWC than WT plants (**Figure 7A**), suggesting that *OsHAK1* overexpression resulted in an increased capacity of water conservation under drought stress. Since Pro has also been suggested to act as an antioxidant to reduce oxidative damage (Székely et al., 2008), the higher Pro in Ox plants may also contribute to some extent, to the lower REL and lower MDA content during drought stress (**Figures 7B,E,F**).

In addition, enhanced activities of antioxidant enzymes, such as SOD, POD, and CAT, are considered a coping strategy for ROS scavenging and reducing programmed cell death in plants (Mittler, 2002; Apel and Hirt, 2004; Farooq et al., 2009). Overexpression of genes involved in ROS detoxification results in lower cellular damage and improved abiotic stress tolerance (Zhang et al., 2013). In *OsHAK1*-Ox plants, increased POX and CAT activity levels (**Figures 8B,C**) and elevated transcription of genes encoding those antioxidant enzymes (**Figures 8D–F**) were detected after drought treatment, indicating that those plants had enhanced capability to scavenge ROS and were better protected from oxidative damage. Consequently, *OsHAK1* overexpression could alleviate oxidative damage in transgenic plants by enhancing Pro accumulation and antioxidant defense.

### *OsHAK1* Activates the Expression of Stress-Responsive Genes

Several reports have established that overexpression of transcription factors leads to induced expression of stress- and ABA-responsive genes, which in turn contributes to improved tolerance to various stresses (Xiong et al., 2014; Zhao et al., 2014; Hong et al., 2016; Jiang et al., 2016; Shen et al., 2017). In our study, expression of six stress-related genes was significantly induced in drought-treated *OsHAK1*-Ox transgenic plants when compared to WT (**Figure 9**). Overexpression of the transcription factor *OsDREB2A* confers salt and dehydration stress tolerance in rice (Cui et al., 2011; Mallikarjuna et al., 2011). SNAC2 is a stress responsive NAC transcription factor that improves cold, salinity, and osmotic stress tolerance of rice (Hu et al., 2008). As discussed, *P5CS1* encodes a key enzyme responsible for Pro synthesis and participates in plant stress tolerance (Yoshiba et al., 1999). *OsbZIP23* is a central regulator in ABA signaling and biosynthesis (Zong et al., 2016) and transgenic rice overexpressing *OsbZIP23* displayed improved tolerance to drought and salinity (Xiang et al., 2008). *OsMYB2*, encodes a stress-responsive MYB transcription factor, is also engaged in rice tolerance to salt, cold, and dehydration stresses (Yang et al., 2012). Overexpression of *AP37* in rice has been reported to enhance tolerance to drought, salinity and low temperature at the vegetative stage and significantly increases grain yield under severe drought conditions (Oh et al.,2009). Therefore, we postulate that the enhanced tolerance to drought stress shown by *OsHAK1* overexpression plants may be explained, in certain degree, by the up-regulation of these genes.

A constant shortcoming caused by constitutive gene over-expression is the abnormal development and often reduced crop productivity (Kasuga et al., 1999; Hsieh et al., 2002; Nakashima et al., 2007; Morran et al., 2011). Therefore, current goals to improve stress tolerance in crops include efforts to diminish any negative impacts on plant growth and yield (Cattivelli et al., 2008). No significant phenotypic differences were observed between *OsHAK1*-Ox lines and control plants under normal conditions (**Figures 3, 4, 10**). Most notably, Ox plants showed significant enhanced drought tolerance in field conditions, with a 35% increase in grain yield per plant over controls (**Figure 10D**). Our data suggest that the *OsHAK1* gene constitute a target for genetic engineering or breeding approaches aiming to the generation of rice cultivars with enhanced drought tolerance.

## Conclusion

**FIGURE 11:**
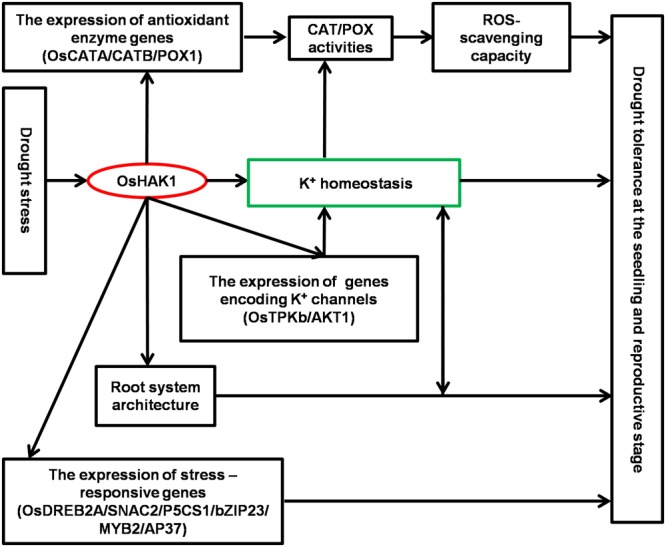
Schematic illustration of potential functions of *OsHAK1* in the regulation of drought tolerance. Drought stress induces the expression of *OsHAK1*, resulting in increased root system and up-regulation of genes encoding K channels to enhance K homeostasis. In addition, there is increased expression of antioxidant enzyme genes implicated in ROS scavenging as a stress defense mechanism and induction of stress-responsive genes to enhance drought tolerance at both the seedling and reproductive stages.

Results of this study demonstrate that *OsHAK1* is a drought-responsive gene which expression is associated to increased dehydration tolerance through the systemic regulation of K homeostasis, root system architecture, Pro accumulation, plasma membrane protection, and activation of stress-related genes (**Figure 11**). Additionally, *OsHAK1* overexpression does not cause any growth defect at the seedling and reproductive stages of plants grown under osmotic and water-limiting conditions, indicating that overexpression of this ion transporter gene is a promising strategy to improve abiotic stress tolerance in cereals. Future investigations can elucidate additional potential functions of *OsHAK1* in response to other abiotic stresses or its interaction with unidentified factors.

## Author Contributions

Conceived and designed the experiments: GC, GX, and QQ. Performed the experiments: GC, CL, YZ, and HJ. Analyzed the data: GC, CL, ZG, GX, and QQ. Contributed reagents/materials/analysis tools: LZ, DR, and LY. Wrote and revised the paper: GC, ZG, GX, and QQ.

## Conflict of Interest Statement

The authors declare that the research was conducted in the absence of any commercial or financial relationships that could be construed as a potential conflict of interest.
