# Supplementary material for: OsHAK1, a High-Affinity Potassium Transporter, Positively Regulates Responses to Drought Stress in Rice
Source: Front Plant Sci. 2017 Nov 1;8:1885. doi: 10.3389/fpls.2017.01885 (PMC5671996; doi:10.3389/fpls.2017.01885)
Supplement: Supplementary file 1 [file Table_1.DOCX]

**Table S1.** Primers for amplifying the *Ubq* and other genes cDNA for Real-Time PCR

| Gene | Primer ID | Primer sequences | |  |
| --- | --- | --- | --- | --- |
| *Ubq* | F(5’-3’) | | ACCCTGGCTGACTACAACATC | |
|  | R(5’-3’) | | AGTTGACAGCCCTAGGGTG | |
| *OsHAK1* | F(5’-3’) | | GTTGATGATGCTGATGTTGGAAG | |
|  | R(5’-3’) | | CCAACACTTTCAGCTGAAAC | |
| *OsTPKb* | F(5’-3’) | | GCTGCACTCGCACACGAT | |
|  | R(5’-3’) | | CCCCGCCGTGTAGAGCTT | |
| *OsAKT1* | F(5’-3’) | | AGAGATCCTTGATTCACTGCC | |
|  | R(5’-3’) | | TCTACTAACTCCACACTACCAG | |
| *OsPOX1* | F(5’-3’) | | CATCCCAGCTCCCAACAA | |
|  | R(5’-3’) | | AGACATGCCAATGGTGTGG | |
| *OsCATA* | F(5’-3’) | | GCCGGATAGACAGGAGAGGT | |
|  | R(5’-3’) | | TCTTCACATGCTTGGCTTCA | |
| *OsCATB* | F(5’-3’) | | GGTGGGTTGATGCTCTCTCA | |
|  | R(5’-3’) | | ATTCCTCCTGGCCGATCTAC | |
| *OsDREB2A* | F(5’-3’) | | GGCTGAGATCCGTGAACCAA | |
|  | R(5’-3’) | | GGACCATACATTGCCCTTGC | |
| *OsSNAC2* | F(5’-3’) | | TGTGCCGGATTTACAACAAG | |
|  | R(5’-3’) | | CACCATCGGCTTCCTCTG | |
| *OsP5CS1* | F(5’-3’) | | GCTGACATGGATATGGCAAAAC | |
|  | R(5’-3’) | | GTAAGGTCTCCATTGCATTGCA | |
| *OsbZIP23* | F(5’-3’) | | GGAGCAGCAAAAGAATGAGG | |
|  | R(5’-3’) | | GGTCTTCAGCTTCACCATCC | |
| *OsMYB2* | F(5’-3’) | | GAGCAGCGAGGAGGAGGT | |
|  | R(5’-3’) | | TGTAGTTGACGAGCAGGAGGT | |
| *OsAP37* | F(5’-3’) | | TCCGATGTTTTGGTCCTCTG | |
|  | R(5’-3’) | | TCCACGGTTTAGTCCATCTCATC | |
