# Supplementary material for: OsHAK1, a High-Affinity Potassium Transporter, Positively Regulates Responses to Drought Stress in Rice
Source: Front Plant Sci. 2017 Nov 1;8:1885. doi: 10.3389/fpls.2017.01885 (PMC5671996; doi:10.3389/fpls.2017.01885)
Supplement: Supplementary file 2 [file Data_Sheet_1.DOCX]

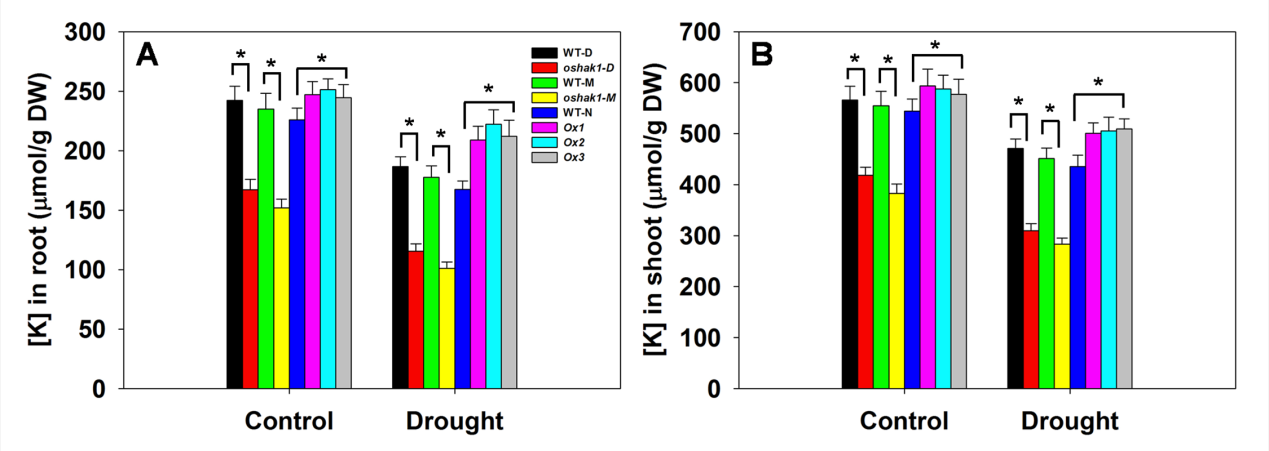


**Supplementary Figure 1.** The effect of *OsHAK1* on K accumulation in plants at the tillering stage challenged by drought stress. [K] in (A) the root and (B) the shoot. Values shown in the form mean±SE (*n=*5). Significant differences (*P*<0.05) between the test genotype and WT are indicated by an asterisk. DW: dry weight.
